# Deciphering Stromal Changes between Metastatic and Non-metastatic Canine Mammary Carcinomas

**DOI:** 10.1007/s10911-023-09542-0

**Published:** 2023-07-01

**Authors:** Julia Ettlin, Alina Bauer, Lennart Opitz, Alexandra Malbon, Enni Markkanen

**Affiliations:** 1 Institute of Veterinary Pharmacology and Toxicology, Vetsuisse Faculty, University of Zurich, Zürich, 8057 Switzerland; 2 Functional Genomics Center Zürich, ETH Zürich/University of Zurich, Zürich, 8057 Switzerland; 3 Institute of Veterinary Pathology, Vetsuisse Faculty, University of Zurich, Zürich, 8057 Switzerland; 4 The Royal (Dick) School of Veterinary Studies and The Roslin Institute, Easter Bush Campus, Midlothian, EH25 9RG Scotland

**Keywords:** Tumour microenvironment, Tumour stroma, Comparative oncology, Dog tumours, Breast cancer malignancy

## Abstract

Cancer-associated stroma (CAS) is widely recognized to influence development and progression of epithelial tumours including breast cancer. Canine mammary tumours (CMTs) such as simple canine mammary carcinomas represent valuable models for human breast cancer also with respect to stromal reprogramming. However, it remains unclear whether and how CAS changes in metastatic tumours compared to non-metastatic ones. To characterize stromal changes between metastatic and non-metastatic CMTs and identify potential drivers of tumour progression, we analysed CAS and matched normal stroma from 16 non-metastatic and 15 metastatic CMTs by RNA-sequencing of microdissected FFPE tissue. We identified 1438 differentially regulated genes between CAS and normal stroma, supporting previous results demonstrating stromal reprogramming in CMTs to be comparable with CAS in human breast cancer and validating deregulation of pathways and genes associated with CAS. Using primary human fibroblasts activated by treatment with TGFβ, we demonstrate some of the strongest expression changes to be conserved in fibroblasts across species. Furthermore, we identify 132 differentially expressed genes between CAS from metastatic and non-metastatic tumours, with strong changes in pathways including chemotaxis, regulation of apoptosis, immune response and TGFβ signalling and validate deregulation of several targets using RT-qPCR. Finally, we identify specific upregulation of *COL6A5*, *F5*, *GALNT3*, *CIT* and *MMP11* in metastatic CAS, suggesting high stromal expression of these targets to be linked to malignancy and metastasis of CMTs. In summary, our data present a resource supporting further research into stromal changes of the mammary gland in relation to metastasis with implications for both canine and human mammary cancer.

**Supplementary Information:**

The online version contains supplementary material available at 10.1007/s10911-023-09542-0.

## Background

Tumour formation is not a strictly cell-autonomous process but results from a reciprocal interaction between the tumour cells and their surrounding tissue, the so-called cancer-associated stroma (CAS) [1, 2]. CAS is composed of a variety of different non-malignant cell types including fibroblasts, adipocytes, immune cells, vascular cells and extracellular matrix (ECM), that provide structure, nutrients and other vital functions to cancer cells. The reprogramming of the normal stromal environment into CAS is strongly driven by the tumour cells through paracrine signalling events and active modulation of the matrix e.g. by proteases. By doing so, cancer cells shape their surroundings into a more favourable habitat to allow for growth, invasion and metastatic dissemination of the diseased cells. Accordingly, current developments aim at targeting this interplay to improve available treatment options for patients and overcome anticancer therapeutic resistance [3]. To date however, our knowledge of the molecular minutiae of stromal changes in patient samples and the molecular dialogue between stroma and the adjacent tumour cells remains incomplete.

Canine simple mammary carcinomas are tumours of the mammary gland that are widely considered to closely mirror human breast cancer and represent valuable models that are not hampered by limitations inherent to rodent models [4–6]. Similar to the situation in women, canine mammary tumours (CMTs) are the most frequent tumours in female dogs [7–9]. In addition to displaying highly comparable biological behaviour, molecular subtypes and common genetic aberrations are conserved to a high degree as well [5, 6]. Importantly, also stromal changes between CMTs and human breast cancer have been shown to exhibit a high degree of cross-species homology, further underlining the validity of CMTs as a model for the human disorder [10–14].

Mammary carcinomas come in different flavours: in women, their formation starts with hyperplastic ductal cells which progress to preinvasive carcinoma in situ (DCIS) and/or invasive breast carcinoma. If these tumours progress further, they can ultimately become metastatic breast cancers, which are advanced tumours that have spread to other organs in the body, such as the draining lymph nodes, the lung, liver bone or brain [15, 16]. The same is suspected at least for a part of mammary tumours in dogs that can originate both from ductal and acinar cells, though the question whether canine carcinoma in situ can be classified as such remains unresolved [17, 18]. As with other tumour types, overall survival of patients with metastatic breast cancer is significantly lower than that of patients without metastases in both species. Increased understanding of mechanisms that drive cancer metastasis is therefore warranted to develop novel therapeutic modalities for these patients.

With its central role in development and progression of mammary carcinomas, CAS is likely to also influence metastasis of tumour cells. Thus far, analyses of stromal reprogramming in human breast cancer have been geared towards understanding differences in normal stroma, DCIS and invasive breast cancer [19–21]. Likewise, we have addressed stromal changes between normal stroma, benign simple mammary adenomas and malignant mammary carcinomas in dogs [10–14]. These studies have clearly shown that the stromal compartment reacts to epithelial changes very early, and that changes in stromal gene expression are strongly driven by the malignancy of the tumour. However, whether and how CAS changes in metastatic compared to non-metastatic tumours of the mammary gland in either species remains unanswered. This question requires addressing to promote development of better therapeutic approaches to treat metastatic breast cancer or even inhibit metastasis formation.

Against this background, we aimed to analyse stromal reprogramming in non-metastatic and metastatic simple canine mammary carcinomas. To do so, we have established a Laser-capture microdissection (LCM) workflow to analyse regions of interest from formalin-fixed paraffin embedded (FFPE) patient tissue by RNA-sequencing or Liquid chromatography-tandem mass spectrometry (LC-MS/MS) [10–13, 22–25]. By applying this approach, we analysed matched CAS and normal stroma from 31 patients with canine simple mammary carcinoma and validated a subset of findings using RT-qPCR. Our results confirm previous reports of stromal reprogramming in CMTs and yield insight into stromal changes of the mammary gland in relation to metastasis with implications for both human and canine patients.

## Results

### Transcriptomic Profiling of Matched CAS and Normal Stroma from 31 Canine Mammary Tumours Isolated by Microdissection of FFPE Specimens

To characterize the difference of gene expression between CAS of CMTs and patient-matched normal stroma from non-cancerous glands, we selected 31 cases of simple mammary carcinomas for which lymph nodes were available to assess presence or absence of metastases (Table 1). Of note, all of the included cases were different from our previously published datasets regarding stromal reprogramming. In 15 of the patients (cases 1–15), metastases could be detected by microscopic tissue examination at the time of tumour excision, whereas no metastases could be found in the other 16 (cases 16–31). All 31 patients were female dogs, and 22 were purebred, 8 crossbreeds and for one dog breed information was not disclosed. The patient age at tumour excision ranged from 5 to 17 years (mean age: 11 years). The age of the FFPE tissue blocks that were used for the study ranged from 7 months to > 120 months based on our experience that sample age does not significantly impact quality of results. Subtype or neutering status was available for 21 of the cases. Tumour grades for metastatic cases were assigned to grade II or III, while non-metastatic cases displayed grades I – III. Clinical follow-up data on survival was available for 10 of the cases (4 metastatic, 6 non-metastatic) for a maximum of 18 months. All 6 non-metastatic cases for which follow-up was available were still alive at 18 months post surgery, while survival in the metastatic cases ranged between 3 and 8 months.

**Table 1:** Overview of simple carcinomas included in this study. Clinical data from patients: Case # = case number as referred to within this study; f = female, intact; f/n = female, neutered; f/n.d. = neutering status not disclosed; Age = patient age at excision of tumour; Subtype = subtype of simple mammary carcinoma if specified; samples marked with “*” were from Berlin and underwent independent review to ascertain simple carcinoma by AM but were not further subtyped. Mets = presence (+)/absence (-) of metastases in lymph nodes; Grade = tumour grade; Sample age = time between initial tumour excision and sampling of stroma/RNA extraction. > 120 denotes samples which are at least 120 months old, but for which no exact age could be defined. Survival = information of survival time after excision of sample in months, where available. Longest time-point of follow-up was 18 months. N.a. = not available

Case #	Gender	Breed	Age (years)	Subtype *not further specified	Mets	Grade	Sample age (months)	Survival (months)
1	f	Volpino Italiano	11	solid	+	III	85	n.a.
2	f	Labrador Retriever	13	comedocarcinoma	+	III	75	n.a.
3	f	Cocker Spaniel	11	solid	+	III	67	n.a.
4	f	n.d.	11	solid	+	III	60	n.a.
5	f	Irish Setter	10	tubulo-papillary	+	II	97	n.a.
6	f/n	Crossbreed	14	solid	+	III	28	n.a.
7	f	Papillon	17	solid	+	III	48	n.a.
8	f/n.d.	American Pitbull	9	*simple carcinoma	+	III	> 120	8
9	f/n.d.	Rottweiler	10	*simple carcinoma	+	III	> 120	6
10	f/n.d.	Bavarian Mountain Hound	13	*simple carcinoma	+	III	> 120	8
11	f/n.d.	West Highland White Terrier	16	*simple carcinoma	+	II	> 120	3
12	f	American Staffordshire Terrier	12	tubular	+	II	59	n.a.
13	f	Border Collie-Mix	14	tubulo-papillary	+	II	80	n.a.
14	f	Vizsla	11	micropapillary	+	II	25	n.a.
15	f	Poodle-Mix	14	comedocarcinoma	+	II	40	n.a.
16	f	Golden Retriever	9	tubular-solid	-	I	15	n.a.
17	f/n.d.	Cocker Spaniel-Mix	12	*simple carcinoma	-	III	> 120	> 18
18	f/n.d.	Labrador Retriever	11	*simple carcinoma	-	III	> 120	> 18
19	f	Old English Sheepdog	11	tubular	-	I	88	n.a.
20	f	Miniature Pinscher	13	tubular	-	I	9	n.a.
21	f/n.d.	Dalmatian	10	*simple carcinoma	-	II	> 120	> 18
22	f/n	Brittany	9	solid	-	II	7	n.a.
23	f/n	Border Collie-Mix	10	tubular	-	II	9	n.a.
24	f/n.d.	German Shepherd-Mix	13	*simple carcinoma	-	III	> 120	> 18
25	f/n.d.	German Shepherd-Mix	9	*simple carcinoma	-	III	> 120	> 18
26	f	Havanese	9	solid	-	II	89	n.a.
27	f/n.d.	Crossbreed	11	*simple carcinoma	-	III	> 120	> 18
28	f	French Bulldog	5	tubular	-	I	21	n.a.
29	f	Staffordshire Bull Terrier	n.d	tubular	-	I	45	n.a.
30	f	Jack Russell Terrier	9	cystic-papillary	-	I	45	n.a.
31	f	Podenco	9	comedocarcinoma	-	I	23	n.a.

**Fig. 1:**
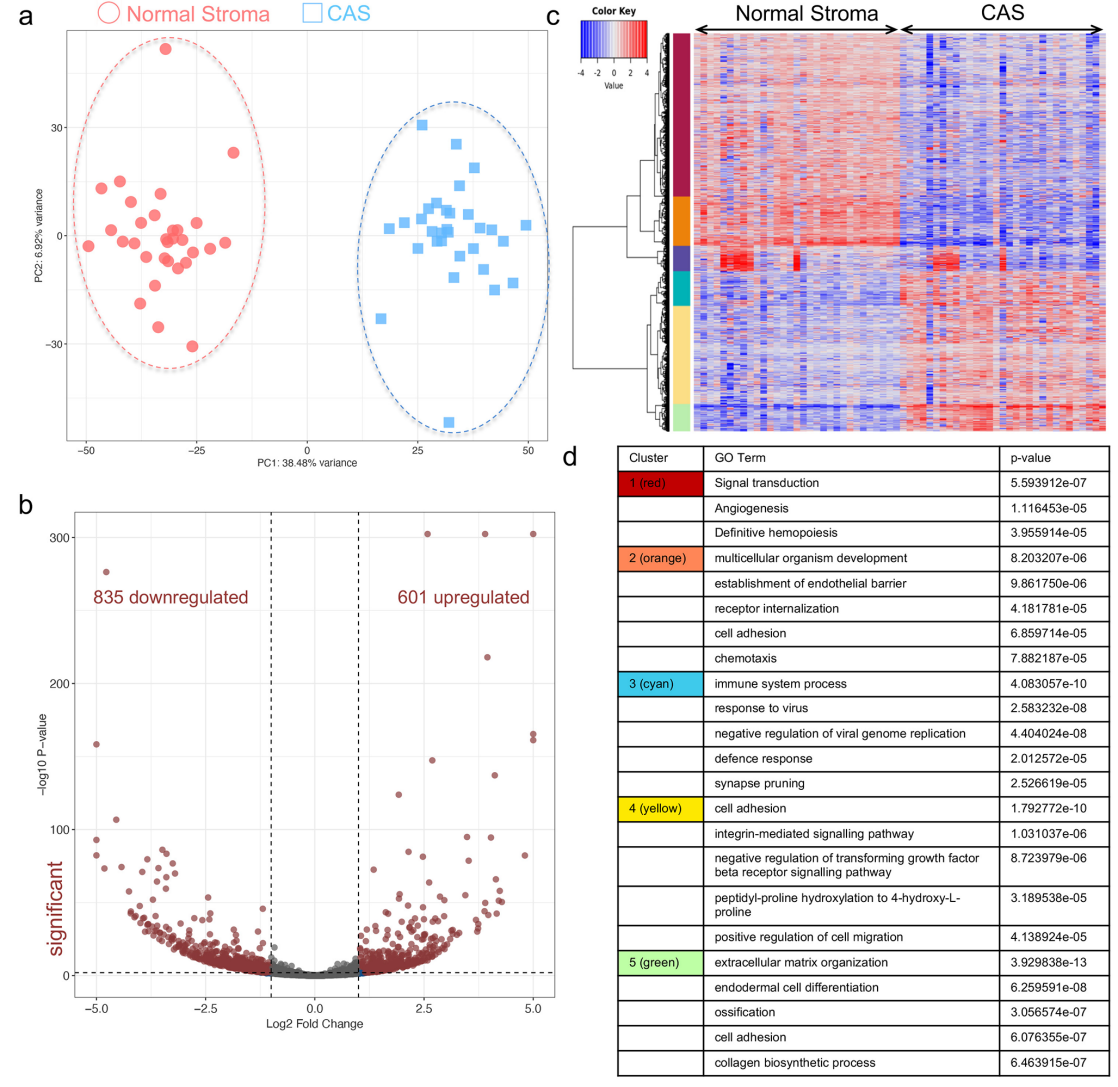
Transcriptomic profiling of matched CAS and normal stroma from 31 canine simple mammary carcinomas. **a**) PCA of CAS and normal stroma samples isolated from 31 cases of simple mammary carcinoma. PCA was performed using all genes. Round red shapes are normal stroma, square blue shapes CAS. **b**) Volcano plot highlighting differentially expressed genes in CAS compared to normal stroma, using fold change > 2 and FDR < 0.01 as cut-off values. **c**) Heatmap and GO analysis for all samples. **d**) Overrepresentation analysis (ORA) of biological processes in the clusters shown as coloured blocks in **c**)

CAS and matched normal stroma (i.e. stroma that is situated next to morphologically unaltered mammary epithelium) was isolated from each of these 31 cases and analysed using our established LCM-RNAseq procedure [10–13, 22–25]. Principal component analysis (PCA) revealed CAS and normal stroma to form 2 clearly distinct clusters, identifying the difference between CAS and normal stroma as the major source of variability in the dataset (Fig. 1a). Furthermore, this demonstrated canine CAS to undergo strong stromal reprogramming, as reported previously [10–13]. Analysis of differentially expressed genes applying a significance threshold of p < 0.01 and a log2 fold-change ≥ 1 to compare CAS and normal stroma identified a total of 1438 genes as significantly differentially expressed, with 601 genes up- and 835 genes downregulated in CAS compared to normal stroma (Fig. 1b, Supplementary Table 1). Gene ontology (GO) analysis identified six clusters (indicated by coloured blocks, Fig. 1c), and overrepresentation analysis (ORA) of biological processes in these clusters identified the following main categories: cluster 1 (red) related to signal transduction, angiogenesis and definitive hemopoiesis, cluster 2 (orange) containing genes involved in multicellular organism development, establishment of endothelial barrier, receptor internalization, cell adhesion and chemotaxis, cluster 3 (cyan) pertaining to immune system process, response to virus, negative regulation of viral genome replication, defence response and synapse pruning, cluster 4 (yellow) exhibited genes involved in cell adhesion, integrin-mediated signalling pathway, negative regulation of transforming growth factor beta receptor signalling pathway, peptidyl-proline hydroxylation to 4-hydroxy-L-proline and positive regulation of cell migration and cluster 5 (green) with genes related to ECM organization, endodermal cell differentiation, ossification, cell adhesion and collagen biosynthetic process (Fig. 1c and d). No GO terms were identified for the blue cluster.

In contrast to human breast cancer, even apparently simple canine mammary carcinomas can contain varying proportions of proliferating myoepithelial cells [17]. If infiltrating into the interstitium, such cells could influence the observed stromal expression patterns. To assess the potential of myoepithelial contamination of our stromal dataset, we analysed expression of typical myoepithelial markers *TP63, CHD3* (P-cadherin), *MYH11, SERPINB5* (Maspin) and *MME* (CD10) [26] in CAS versus normal stroma. While expression of *ACTA2* – a marker expressed by both myoepithelial cells and cancer-associated fibroblasts (CAFs) – significantly increased in CAS compared to normal stroma, expression of *TP63, CHD3, MYH11*, *SERPINB5* and *MME* did not increase (Supplementary Fig. 1a). Moreover, comparing expression of ACTA2 between the cases that were p63-immunohistochemistry negative to all cases reveals no difference with respect to expression levels (Supplementary Fig. 1b). Together, these findings suggest low to negligible contamination with myoepithelial cells in this stromal dataset.

**Fig. 2:**
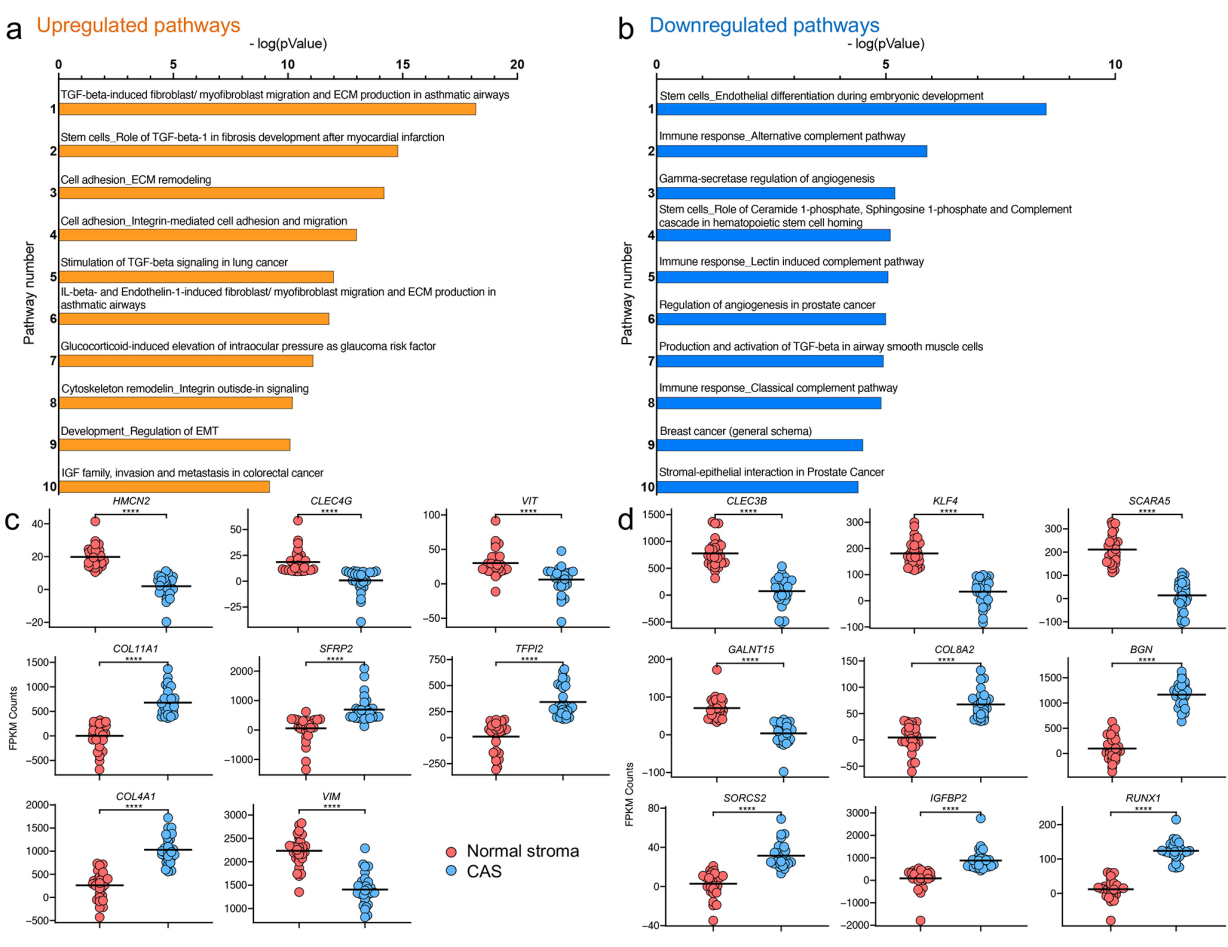
Analysis of differentially expressed genes in CAS vs. normal stroma validates previous findings of stromal reprogramming in CMTs. **a** and **b**) Pathway analysis of genes upregulated (**a**) and downregulated (**b**) in CAS vs. normal stroma. The x-axis shows the -log(pValue) describing the extent of the intersection between the stromal genes from CAS and normal stroma and all the genes involved in each pathway. The y-axis displays the 10 most significant pathways. **c**) Validation of key findings from [11] using the new dataset. **d**) Validation of key findings from [12] using the new dataset

To gain further insight into the deregulated pathways between CAS and normal stroma, we applied the MetaCore™ program to analyse the 500 most deregulated genes with pathway maps setting a threshold of 0.5 and p-value of 0.05. The upregulated genes were dominated by Transforming growth factor β (TGFβ) signalling, cell adhesion and ECM remodelling/integrin-mediated cell adhesion and migration, interleukin beta and endothelin-1 signalling and regulation of epithelial-to-mesenchymal transition (EMT) among the top 10 pathways. In contrast, angiogenesis, immune response and the complement pathway and stem cell/differentiation processes were present among the top 10 pathways of the downregulated genes (Fig. 2a and b). As such, these results are consistent with changes in stromal biology, further validating our analytic approach to assess reprogramming in the stromal compartment of patient samples.

We have previously reported strong stromal remodelling in 13 canine mammary adenomas and 15 carcinomas [11, 12, 14]. To understand whether some of the key changes observed in these studies could be verified in this independent and larger cohort of patients, we assessed expression of genes highlighted in these studies in this new dataset. Consistent with findings reported in [11], we found a significant decrease in *HMCN2, CLEC4G*, *VIT* and *VIM* expression, whereas *COL11A1*, *SFRP2*, *TFPI2* and *COL4A1* significantly increased in CAS compared to normal stroma (Fig. 2c). Furthermore, in line with results reported in [12], *CLEC3B, KLF4, SCARA5* and *GALNTT15* were downregulated while *COL8A2, BGN, SORCS2, IGFBP2* and *RUNX1* were upregulated in CAS vs. normal stroma (Fig. 2d). As such, these results validate previous reports of stromal changes in CMTs and significantly extends the available data for stromal reprogramming in these canine mammary carcinomas to a total of 46 patients.

### Prominent Stromal Changes are Conserved in Primary Human Fibroblasts Activated with TGFβ

**Fig. 3:**
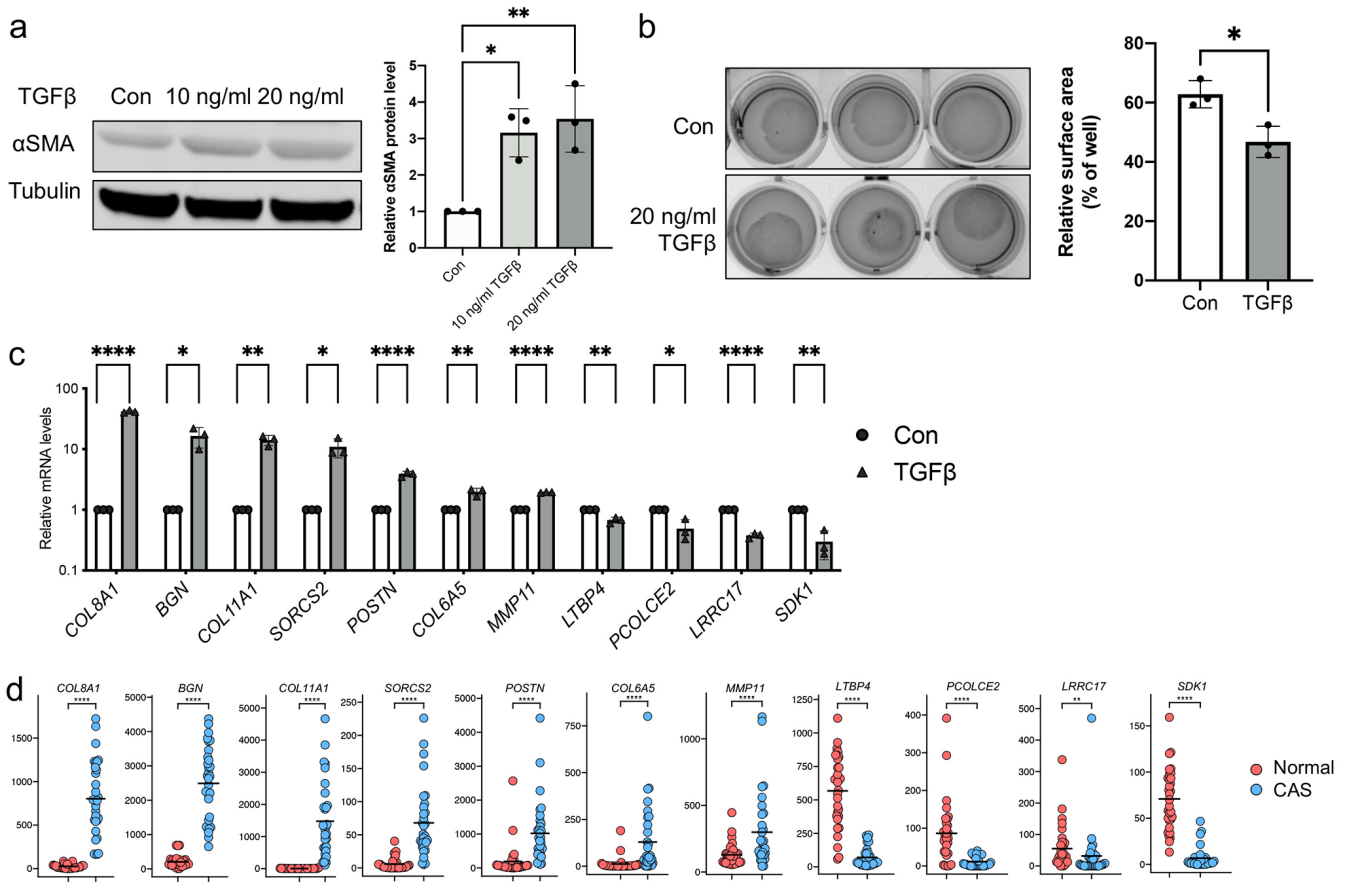
Changes in canine CAS are mirrored in primary human fibroblasts activated with TGFβ. **a**) Representative Western blot (left) and quantification of relative α-SMA protein levels (right) of Tig-1 human primary fibroblasts activated with TGFβ using α-SMA and tubulin antibodies. **b**) Collagen contraction assay (left) of Tig-1 human primary fibroblasts activated with TGFβ and quantification of relative surface area (right). **c**) Relative mRNA levels of *COL8A1, BGN, COL11A1, SORCS2, POSTN, COL6A5, MMP11, LTBP4, PCOLCE2, LRRC17* and *SDK1* in Tig-1 human primary fibroblasts activated with TGFβ as assessed by RT-qPCR. **d**) TPM counts of the targets shown in **c**) as detected in normal stroma and CAS by RNA-sequencing

In line with the situation in human breast cancer, we have previously identified fibroblasts as the dominant cell type in CAS of CMTs [12]. Given the strong TGFβ-related signature that emerged from the above analyses and TGFβ’s central role in conversion of fibroblasts to myofibroblasts or CAFs, we hypothesized that similar changes could be elicited in primary human fibroblasts by treating them with TGFβ [27, 28]. To this end we exposed Tig-1 primary human fibroblasts grown in culture dishes to TGFβ or vehicle control for 72 h and assessed them using Western blot and a collagen contraction assay. As expected, treatment with TGFβ resulted in an activation of Tig-1 towards a myofibroblastic phenotype, as evidenced by a strong increase in protein levels of α smooth muscle actin (αSMA) as well as enhanced contractility compared to control (Fig. 3a and b). Subsequently, a selection of fibroblast-related genes that were significantly deregulated in our normal vs. CAS data was analysed using quantitative real-time PCR (RT-qPCR). Interestingly, we could verify significant increases in transcription of *COL8A1, BGN, COL11A1, SORCS2, POSTN, COL6A5* and *MMP11* and significant downregulation in *LTBP4, PCOLCE2, LRRC17* and *SDK1* upon activation of Tig-1 cells with TGFβ compared to control treatment (Fig. 3c). These changes were consistent with changes between canine CAS and normal stroma as observed by RNAseq (Fig. 3d). The conservation of the fibroblast-related stromal changes observed in CMTs in activated human fibroblasts underlines the contribution of fibroblasts to the transcriptional changes observed in CAS of CMT and suggest molecular similarity between canine and human stromal reactions.

### Stromal Differences Between Metastatic and non-metastatic Canine Mammary Tumours

**Fig. 4:**
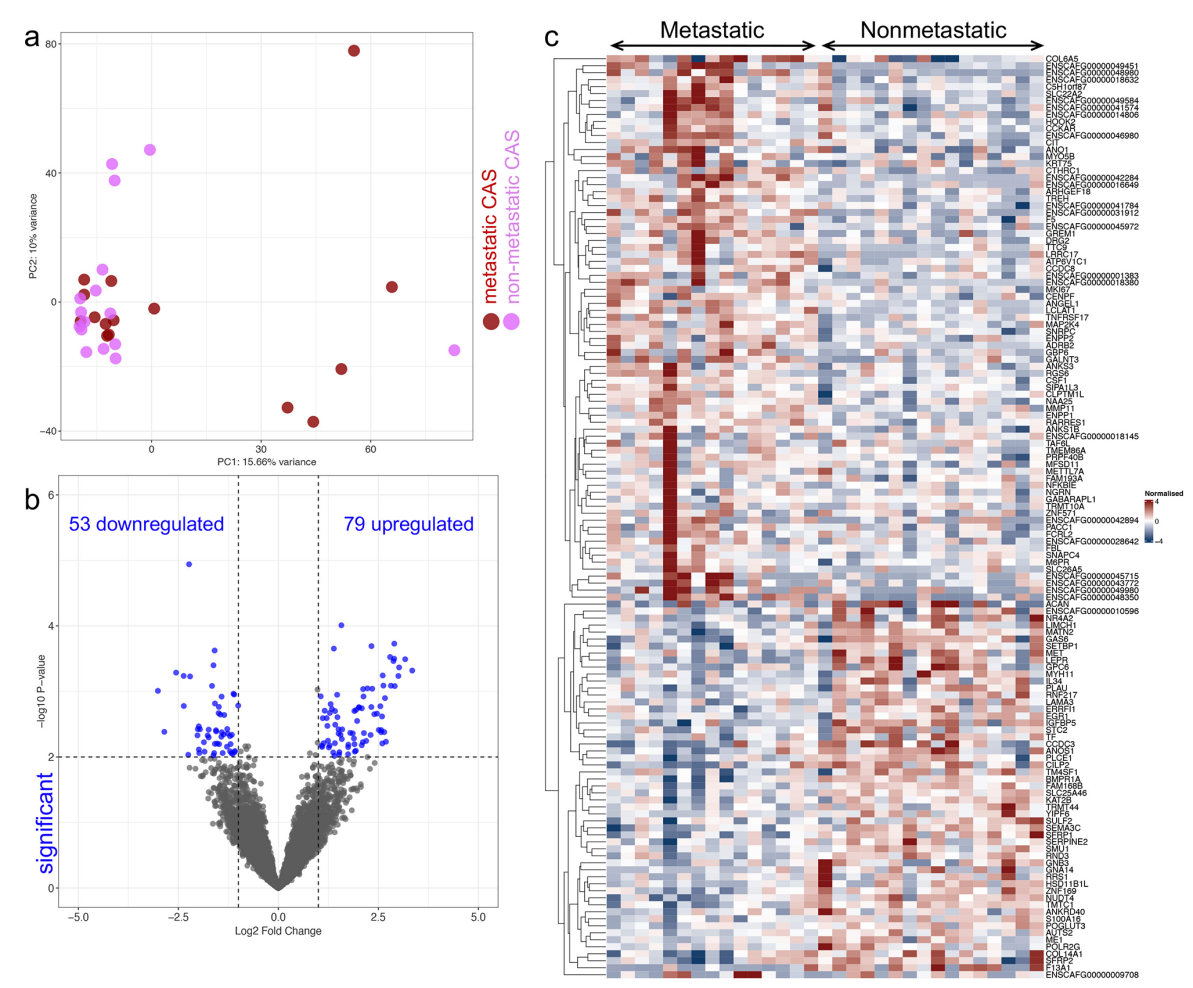
Transcriptional changes in CAS between metastatic and non-metastatic canine mammary carcinomas. **a**) PCA of CAS and normal stroma of the comparison metastatic and non-metastatic samples isolated from all cases. PCA was performed using all genes. **b**) Volcano plot highlighting differentially expressed genes in metastatic vs. non-metastatic CAS compared, using fold change > 2 and p ≤ 0.01 as cut-off values. The number of significantly deregulated genes is indicated. **c**) Heatmap of significantly deregulated genes from **b**) in CAS of metastatic (left) and non-metastatic (right) cases. Each row features one gene, and each column represents one sample. The red and blue colours represent the relative gene expression level of each gene for each sample in relation to all the other samples. Red indicates a relative up-regulation and blue indicates a relative down-regulation of the gene

Next, we aimed to address whether and how stroma changes in relation to tumour metastasis. To this end, we compared stromal gene expression of metastatic vs. non-metastatic CMTs. PCA did not distinguish between metastatic and non-metastatic CMTs using the first two components, indicating changes between the two conditions to be more subtle than between normal stroma and CAS, as would be expected (Fig. 4a). To identify differentially expressed genes between the two conditions, we compared gene expression between metastatic and non-metastatic CMTs applying a significance threshold of p < 0.01 and log2 ratio ≥ 1. By doing so, we identified 132 differentially expressed genes between CAS from metastatic vs. non-metastatic samples, including 79 upregulated genes and 53 downregulated genes in the metastatic group compared to the non-metastatic group (Fig. 4b and c and Supplementary Table 2).

**Fig. 5:**
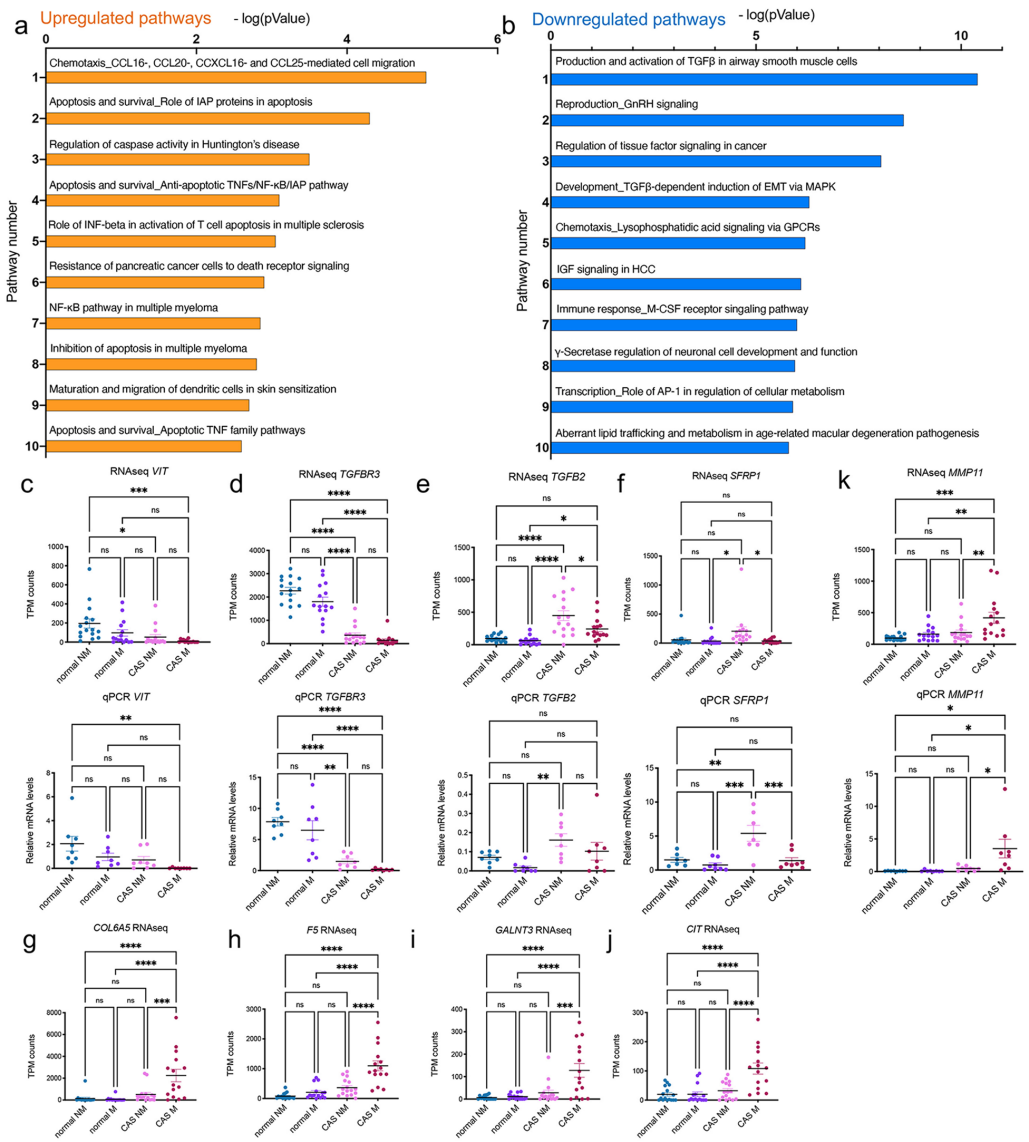
Identification of targets selectively upregulated in CAS from metastatic mammary carcinomas. **a** and **b**) Pathway analysis of genes upregulated (**a**) and downregulated (**b**) in CAS from metastatic compared to non-metastatic samples. The X-axis shows the -log(pValue) describing the extent of the intersection between the stromal genes from metastatic tumours and all the genes involved in each pathway. The Y-axis displays the 10 most significant pathways. c – f) RT-qPCR validation of selected genes from the RNAseq dataset. Relative mRNA levels of stromal genes in CAS of non-metastatic tumours (CAS NM), CAS of metastatic (CAS M) tumours and respective normal stroma (normal NM and M), measured by RNAseq (top) and validated by RT-qPCR (bottom). Scatter plots for c: *VIT*, d: *TGFBR3*, e: *TGFB2*, and f: *SFRP1*. The value of each sample is displayed for each condition with a mean value ± SEM. Significance between the different conditions was calculated using ANOVA followed by Bonferroni’s Multiple Comparison Test to compare all conditions with each other, and is indicated with * = p < 0.05, ** = p < 0.01, *** = p < 0.001, **** = p < 0.0001 and ns = not significant, respectively. n = 15 metastatic, 16 non-metastatic sample pairs for RNAseq, n = 8 for metastatic and 8 for non-metastatic sample pairs for RT-qPCR. g – k) RNAseq values of genes specifically upregulated only in metastatic CAS displayed as in c-f. g) *COL6A5*, h) *F5*, i) *GALNT3*, j) *CIT*, k) *MMP11* and RT-qPCR validation of *MMP11*

Gene set enrichment analysis identified changes in genes related to the extracellular region (GO:0005576, enrichment score − 0.397) and extracellular space (GO:0005615, enrichment score − 0.313), respectively while ORA of GO biological processes revealed significant enrichment in G protein-coupled receptor signalling among the upregulated genes (p = 0.0245), and chrondrocyte development (p = 0.0000610), negative regulation of gene expression (p = 0.0000738), somitogenesis (p = 0.000369), outflow tract morphogenesis (p = 0.000369) and cartilage development (p = 0.000925) among the downregulated genes. To gain more insight into the changes between non-metastatic and metastatic tumours, the 500 most deregulated genes were analysed using MetaCore™ with a threshold set at 0.5 and p-value of 0.03. Pathway analysis of the upregulated genes in the CAS of metastatic tumours showed involvement in chemotactic cell migration, regulation and inhibition of apoptosis and survival as well as activation of the immune response (Fig. 5a). The downregulated genes in the metastatic samples were characterized by TGFβ signalling, gonadotropin-releasing hormone (GnRH) signalling, tissue factor signalling, and genes involved in immune response and lipid metabolism among the top 10 deregulated pathways (Fig. 5b).

To validate some of the gene expression changes detected, we performed RT-qPCR with 16 randomly selected cases (8 metastatic (M), 8 non-metastatic (NM)) for 4 selected genes. These included *VIT*, *TGFBR3*, *TGFB2*, and *SFRP1*. Overall, the expression trends were highly comparable between RNAseq and RT-qPCR (Fig. 5c – f, RNAseq on top, qPCR in bottom). For *VIT*, RNAseq indicated significant changes between normal NM and both CAS subtypes, while RT-qPCR results only reached significance between normal NM and CAS M samples (Fig. 5c). *TGFBR3* expression consistently showed significant differences between normal stroma and CAS, both in RNAseq and RT-qPCR analysis (Fig. 5d). RNA levels of *TGFB2* significantly increased in CAS of non-metastatic tumours more than metastatic ones by RNAseq, a trend that was mirrored by RT-qPCR analysis (Fig. 5e). Similarly, *SFRP1* levels in non-metastatic CAS were significantly higher than in normal stroma or metastatic CAS, respectively (Fig. 5f). Finally, we were interested in identifying targets significantly upregulated in metastatic CAS but not changing in the other three conditions. Manual curation of the list of differentially expressed genes revealed *COL6A5, F5, GALNT3, CIT* and *MMP11* to meet these criteria (Fig. 5g - k). The specific increase in *MMP11* expression in metastatic CAS was further validated by RT-qPCR (Fig. 5k). Hence, high stromal expression of these 5 targets seems to be strongly linked to malignancy and metastasis of CMTs.

## Discussion

CAS is well-established to hold a key role in initiation and progression of human breast cancer [1, 2]. Accordingly, there is significant interest in understanding how stromal reactions differ between benign and malignant forms of the disease with the goal to gain mechanistic insight into stromal determinants of tumour malignancy. While previous studies have demonstrated stromal changes to predict clinical outcome in human breast cancer [19, 20, 29, 30], there are no comparable datasets available for CMTs. Considering CMTs have been demonstrated to present valuable models for human breast cancer both with regards to genomic characteristics of tumour cells as well as stromal reactions to neoplastic growth [5, 11–14], the question whether and what stromal changes occur in metastatic compared to non-metastatic CMTs becomes highly relevant not only to advance canine healthcare, but also from a cross-species point-of-view. Here we significantly extend the currently available data on stromal reprogramming in CMTs by analysing a total of 31 matched pairs of normal stroma and CAS using LCM-RNAseq and dissect differences between CAS of metastatic and non-metastatic CMTs. As expected, CAS clearly differred from normal stroma (Fig. 1), supporting our previous reports of extensive stromal reprogramming in CMTs [11, 12]. Interestingly, 7 of the 10 top upregulated pathways detected were also among the top 10 pathways in our proteomic study of CAS vs. normal stroma using LC-MS/MS [13]. Overall, the observed changes are consistent with remodelling that is driven by both fibroblasts and immune cells, both of which are key components in the development of breast cancer [1, 31]. Alterations in the immune response and emergence of tumour-promoting inflammations is a hallmark of tumours that has gained a lot of attention over the last decade [2]. In line with our findings, increased numbers of macrophages in tumours have been shown to be associated with more aggressive features in CMTs [32]. Of note, many changes pertaining to tumour-associated inflammation are conserved between CMTs and human breast cancer [33]. Further to this, immune escape has also been found to constitute an integral part of the transition from DCIS to invasive ductal carcinoma in humans, suggesting the potential for immunotherapies in treating these tumours [34]. Interestingly, cross-reactivity and functionality of approved human immune checkpoint blockers have been assessed in dogs [35]. Hence, given the inherent problems with classical rodent-based preclinical models to accurately recapitulate the immune microenvironment of human breast tumours, CMTs would be well positioned to help advance these investigations.

Of note, the aim of the study was to include only simple carcinomas, excluding tumours with a proliferating myoepithelial component. As H & E based assessment can underestimate the presence of malignant myoepithelial cells [17], and p63 staining for myoepithelial was only performed in cases that were not clear based on H & E, we cannot completely rule out that some of the included cases might contain a myoepithelial component, which could also influence gene expression results in canine CAS. However, assessment of typical myoepithelial markers that are considered not or very lowly expressed by myofibroblasts present in CAS and highly comparable ACTA2 expression between confirmed p63 negative and all cases suggest low to negligible contamination with myoepithelial cells in this stromal dataset (Supplementary Fig. 1). Hence, while still possible, we expect potential myoepithelial contamination to be minimal and therefore not to strongly impact on our results. This notion is further supported by our expression data, which clearly clusters CAS from all cases (Fig. 1a), and the fact that the expression changes overall are highly comparable to our previously published data from canine simple mammary carcinomas (Fig. 2). Finally, the fact that highly comparable stromal reprogramming is well documented in human breast cancer, which are less likely to contain a neoplastic myoepithelial component, further supports this interpretation.

As the most abundant cell type of the stroma, the main function of fibroblasts is the production and maintenance of ECM [36]. They are reactive cells characterized by a high degree of plasticity and rapidly adapt to changes in their surroundings by altering their phenotypic, contractile and secretory properties. When in the vicinity of tumour cells, this plasticity leads to reprogramming of fibroblasts towards CAFs, which are known to exert manifold tumour-supporting and promoting functions, including therapeutic resistance of tumours [2, 36]. Using human primary fibroblasts that were activated using TGFβ treatment towards a CAF-like phenotype in vitro [27, 28, 37], we could demonstrate some of the most prominent fibroblast-related changes observed in CAS from CMT to be conserved between the canine CAS and human fibroblasts (Fig. 3). Importantly, increases in *COL8A1*, *BGN, COL11A1, POSTN, MMP11* and *SORCS2* and decreased *LTBP4, PCOLCE2, LRRC17* and *SDK1* have been identified in CAS and CAFs from human breast cancer and are consistently perturbed in stroma of canine and human mammary cancer [11, 19, 20, 30, 38–42]. Furthermore, we have shown expression of *COL8A1, COL11A1, BGN, SORCS2, POSTN* and *COL6A5* to be increased in CAS of malignant CMTs compared to benign adenomas on both the RNA and protein level, and high expression of *COL6A5* and *POSTN* to correlate with significantly lower overall survival in human breast cancer patients [13]. Hence, these results highlight the contribution of fibroblasts to the changes observed in CAS of CMT, further supporting the notion of molecular homology between canine and human stromal reactions and the potential value of the canine model for human breast cancer.

Our analysis of stromal changes between non-metastatic and metastatic canine mammary carcinomas highlighted molecular differences in the tumour microenvironment, including changes in *VIT, TGFBR2, TGFBR3, LTBP4* and *SFRP1* (Figs. 4 and 5). We have previously reported a progressive decrease of *VIT*, a gene involved in remodelling of the ECM, in CAS of malignant CMTs compared to benign adenomas on both the RNA and protein level, and high expression of *VIT* to correlate with significantly better overall survival in human breast cancer patients [12]. Both *TGFB2* and *TGFBR3* are directly involved in TGFβ signalling, which regulates many different aspects of tumour formation and progression. Of note, *LTBP4*, a regulator of TGFβ signalling, was also found to decrease in CAS vs. normal stroma and in fibroblasts activated using TGFβ treatment (see above). One study found down-regulation of various growth factors, including their receptors like *TGFBR2* or *TGFBR3* in metastatic CMTs when comparing with normal tissue [43]. Moreover, decreased expression of *TGFBR3* was associated with malignancy in various cancers [44–46]. Expression of *SFRP1* has been previously reported to be strongly downregulated in invasive breast carcinomas, though in contrast to our stromal dataset these studies focused on the epithelial expression of *SFRP1* [38, 47]. Given its role as a negative regulator of the WNT-pathway it is tempting to speculate *SFRP1* expression to be increased in the stroma as a protective reaction in non-metastatic tumours against EMT induction, whereas in the absence of such an increase EMT can promote metastatic dissemination of tumour cells. This is in line with a recent report that high *SFRP1* expression was related to favourable long-term survival in breast cancer patients [48]. Furthermore, we have found *SFRP1* expression to increase in the stroma of canine mammary adenomas, suggesting *SFRP1* expression to be an early stromal reaction to epithelial hyperplasia [12].

Finally, in an attempt of identifying targets that are specifically upregulated in metastatic stroma, we identify 5 highly interesting genes, including *COL6A5*, *F5*, *GALNT3*, *CIT* and *MMP11* (Fig. 5g-k). Importantly, specific upregulation of *MMP11* in CAS of metastatic tumours was validated by qPCR (Fig. 5k). As such, high stromal expression of these 5 targets seems to be strongly linked to malignancy and metastasis of CMTs. The relation between *COL6A5* expression and malignancy is further supported by the fact that *COL6A5* expression was not changing between normal stroma and benign canine mammary adenomas, but showed high expression in canine mammary carcinomas and correlates with worse overall survival in human breast cancer patients [13]. In line with the identification of *F5*, an essential cofactor in blood coagulation, as significantly elevated in metastatic stroma, we have reported its expression to progressively increase from canine mammary normal stroma, benign adenoma and carcinoma [11, 12]. Moreover, high expression of *F5* in human breast tumours has been linked to tumour aggressiveness and overall survival [49]. Indeed, coagulation is thought to promote growth of tumours and new vasculature [50]. Until now however the mechanistic aspects by which stromal *F5* could impact tumours have not been addressed. *GALNT3* is involved in posttranslational modification of *FGF23*, which in turn regulates phosphate reabsorption by the kidneys [51]. Thus far, there are no reported connections between *GALNT3* and CIT expression and breast cancer. Given that *GALNT3* has also been found to be expressed in certain immune cells, it is conceivable that the increase in *GALNT3* detected in metastatic tumours reflects changes in immune components of the stroma [52]. There is abundant literature assessing the link between *MMP11* and breast cancer. *MMP11* is a matrix metalloproteinase which, in contrast to other MMPs, does not have any direct influence on the degradation of the ECM but cleaves enzymes including proteinase inhibitors [53, 54]. Originally detected as a protein specifically expressed in stromal cells of invasive breast carcinomas [55], expression of *MMP11* in breast cancer stroma has been associated with higher risk of invasive tumour growth and to correlate with worse clinical outcome in patients with invasive breast cancer, thereby potentially serving as prognostic factor [53, 54, 56, 57]. *MMP11* was also increased in both the stroma of mammary carcinomas [11], in whole tumours of metastatic canine mammary carcinomas compared to non-metastatic ones [43], and its expression was correlated with invasiveness in human breast cancer [19, 58]. Interestingly, *MMP11* has been shown to be expressed both by CAFs, adipocytes as well as mononuclear inflammatory cells in breast cancer and to be significantly correlated with immune cell infiltration [59, 60]. Indeed, in gain-of-function and loss-of-function experiments with mice, *MMP11* has been shown to favour early tumour growth by boosting proliferation of cells and reducing their apoptosis by promoting metabolic flexibility that promotes tumour cell growth [61]. The exact molecular mechanism by which *MMP11* elicits these changes, and whether its catalytic activity is required for the full effect remains to be determined. A limitation in our study is that clinical follow-up data on survival was only available for 10 of the investigated cases (Table 1). Based on the available survival data, all 6 dogs of the non-metastatic group survived > 18 months after excision of the tumour, while the 4 cases classified as metastatic had very short survival (3–8 months). While we acknowledge the shortcoming regarding our follow-up data, it has been shown that presence of metastases in lymph nodes are negatively associated with overall survival, as would be expected from a biological point of view (e.g. [62–64].). Hence, while we cannot exclude that some of the cases labelled ‘non-metastatic’ might have developed regional or distant metastases at a later time-point, the fact that no metastases were present at the time of excision certainly argues for less malignant or advanced disease. Moreover, as we assess the difference between the metastatic group (certain metastatic) and the non-metastatic group (from which a few cases might go on later to develop metastases all the same), our results are bound to underplay the difference between the groups mitigating the risk of overinterpretation.

Concluding, the data presented in this paper extends our knowledge regarding stromal reprogramming in CMTs, yields valuable insight into the stromal changes associated with tumour metastasis of CMTs and identify several interesting, deregulated targets, suggesting that stromal changes could potentially be used as markers for tumour progression. Provided more mechanistic understanding of the role and effect of these changes on tumour metastasis, some of the observed changes might present therapeutic targets to prevent spreading of tumour cells. Finally, given the high degree of cross-species molecular homology with respect to stromal reprogramming in tumours of the mammary gland between humans and dogs, these findings have the potential to further support the understanding human breast cancer from the viewpoint of comparative oncology.

### Ethics Approval and Consent to Participate

No animals were killed for the purpose of this research project, as the tissue analysed had been surgically removed in a curative setting with the verbal consent of the patient owners. According to the Swiss Animal Welfare Law Art. 3 c, Abs. 4 the preparation of tissues in the context of agricultural production, diagnostic or curative operations on the animal or for determining the health status of animal populations is not considered an animal experiment and, thus, does not require an animal experimentation license. All the used FFPE specimen were obtained for diagnostic reasons and do therefore not require a formal ethics approval, in full compliance with national guidelines.

## Methods

### Selection of Cases and Tissue Processing for LCM

Twenty-one canine simple mammary carcinomas were provided by the Institute of Veterinary Pathology of the Vetsuisse Faculty Zurich. These represent cases that were either from the Small Animal Hospital of Zurich or external cases sent in by Swiss veterinarians. An additional ten cases were provided by Prof. R. Klopfleisch from the Institute of Veterinary Pathology of the Freie Universität Berlin and have been part of a previous study [43]. All samples were formalin-fixed, paraffin embedded (FFPE) were selected and independently reviewed (including cases from Berlin) by a board-certified veterinary pathologist (AM). The criteria for inclusion in this study were as follows: female dogs, simple mammary carcinomas, histological tumour grade I-III, sufficient tumour and normal stroma for isolation, available information on lymph nodes regarding metastases. The cases were reviewed using routinely stained H & E slides cut at 2 μm and were classified according to Goldschmidt et al. 2011. If no clear diagnosis of sub-type was reached on routine histological assessment, the cases were first discussed with colleagues of the institute. In unclear cases, differentiation between simple and complex carcinomas was achieved using immunohistochemistry for p63 (1:50, Abcam #ab735) to detect myoepithelial cell proliferation, according to a standard protocol. Cases that showed p63 positive proliferations/aggregates of myoepithelial cells were excluded from further analysis. Nevertheless, it is possible that some of the included cases that were judged upon H & E staining alone also contain proliferating myoepithelial cells, as these cannot always be excluded by H & E alone [17]. Tissue processing was performed as described in [22]. Table 1 provides an overview of all cases included in the study.

### Laser-capture Microdissection (LCM)

Sections were stained using Cresyl Violet according to [11] and reviewed by a veterinary pathologist before microdissection to identify the stromal areas. Cell types included for isolation were fibroblasts, endothelial cells, pericytes, and inflammatory cells. Normal stroma was isolated from the same slides as the tumour-associated stroma according to well-established criteria [10–12, 20]: normal stroma was only considered if located *at least* 5 mm away from tumour cells, located between unaltered mammary epithelia and devoid of any obvious alterations such as heavy inflammation or similar. Due to the patient-matched design of the study potential influences that might affect the entire animal, such as hormonal status or similar were not considered, as they would be expected to influence both CAS and normal stroma at the same time. If the sample did not meet the criteria for normal stroma, normal stroma was isolated from another mammary tissue specimen of the same dog, extracted and fixed on the same day. Laser-capture microdissection was performed using the ArcturusXT™ Laser Capture Microdissection System (Thermo Scientific) and the Arcturus® CapSureⓇ Macro LCM Caps (Life Technologies) as detailed in [10, 13]. Isolation of areas of interest was verified by microscopic examination of the LCM cap as well as the excised region after microdissection. After excision, the filled caps containing tissue were put on a 1.5 ml centrifuge tube (EppendorfⓇSafe-Lock tubes) and frozen at − 20 °C until RNA extraction.

### RNA Isolation

RNA was isolated using the Covaris truXTRAC FFPE RNA kit and the Covaris E220 focused ultrasonicator as described in [11]. Details about RNA concentration, yield, and quality for all samples can be found in Supplementary Table 3 RNAvalues.

### RNA Sequencing

2.5 ng totalRNA per sample was used for library preparation with the SMARTer Stranded Total RNA-Seq Kit v2– Pico Input Mammalian (Clontech, Takara Bio). The final libraries were loaded on a HiSeq 4000 (Illumina) and sequenced in single read 125 nt mode.

### Bioinformatics Analyses

The raw reads were first cleaned by removing adapter sequences, trimming low quality ends, and filtering reads with low quality (phred quality < 20) using Fastp (Version 0.20) [65]. Sequence pseudo alignment of the resulting high-quality reads to the Canine reference genome (build CanFam3.1, gene model definitions based on Ensembl release 104 downloaded on 06/01/21) and quantification of gene level expression was carried out using Kallisto (Version 0.46.1) [66]. Differential expression was computed using the generalized linear model implemented in the Bioconductor package edgeR (R version: 4.2.0, edgeR version: 3.38.1) [67]. Data normalization was performed with the TMM method and p-value were adjusted with the Benjamini and Hochberg method. The sequencing raw data was submitted to ENA and is available under the accession id PRJEB57447.

### RT-qPCR of Patient-derived RNA Samples

Quantitative RT-PCR using Taqman primers was performed as described in [22] using 16 randomly selected cases (8 metastatic, 8 non-metastatic). Primers are detailed in Supplementary Table 4.

### Cell Culture

Tig-1 primary human fibroblasts were purchased from Coriell and cultured under standard conditions (37 °C, 5% CO2) in Gibco™ DMEM, low glucose, GlutaMAX™ Supplement, pyruvate, containing 15% foetal calf serum (FCS). 24 h before treatment, 500,000 cells were seeded onto 10 cm dishes. For treatment with human TGFβ (Sigma-Aldrich, #T7039-50UG), fresh medium (DMEM 15% FCS) supplemented with recombinant protein was added at indicated concentrations and incubated for 72 h until further processing.

### Western Blot

Cells were harvested using a cell scraper in cold 1 x PBS and pelleted by centrifugation. Cell lysates were prepared by adding 100 µl of M-PER mammalian protein extraction reagent (Thermo Scientific, #78,501) supplemented with cOmplete, EDTA-free Protease Inhibitor (Roche #11,873,580,001) and incubating for 30 min on a rotating wheel at 4 °C. This was followed by 3 cycles of sonication (30 s on, 30 s off) in a sonication water bath @ 4 °C. Cell debris was pelleted by centrifugation for 15 min at maximal speed and the soluble supernatant was transferred into a fresh Eppendorf tube. 40 µg of total protein per sample was separated on a 4–20% Tris-Glycine gel (Novex) and transferred onto Immobilon-FL polyvinylidene fluoride (PVDF) membranes (Millipore) according to standard procedures (Novex). After blocking overnight, the membrane was probed for 2 h with primary antibodies for αSMA (mouse monoclonal, DakoCytomation, #M0851) and α-tubulin (mouse monoclonal, Sigma, #T5168-100UL) followed by 1 h with a secondary antibody conjugated with Alexa Fluor IRDye 800CW (Li-cor Biosciences). Detection and quantification was performed using the OdysseyCLX image analysis system (Li-cor Biosciences). Tubulin served as the loading control. Each experiment was independently repeated three times. For quantification, protein levels were first normalized to the loading control and then to the respective control lane.

### RT-qPCR of Tig-1 Cells

RNA from untreated and TGFβ treated (20ng/ml) fibroblasts was extracted with the RNeasy Mini Kit (Qiagen) following the manufacturer’s protocol. For homogenization of the lysate the method with needle and syringe was chosen. Equal amounts of RNA were reverse transcribed using the iScript cDNA Synthesis Kit (Biorad). Quantitative real-time PCR (RT-qPCR) was performed using the KAPA SYBR® FAST One-Step qRT-PCR Kit in a total volume of 10 µl in duplicates on the CFX384 Touch™Real-Time PCR detection system (Bio-Rad). For quantification of gene expression the comparative CT method was applied. Values were normalised against GAPDH and B2M, followed by the control, and results were expressed as fold change in mRNA levels over control cells. Each experiment was independently repeated three times. Primers are detailed in Supplementary Tables 4 and were ordered from Microsynth (Balgach).

### Collagen Contraction Assay

Untreated and TGFβ (20ng/ml) treated Tig-1 cells were harvested with 2.5% trypsin, neutralised using defined trypsin inhibitor (1x) (Gibco, #R007100), and diluted in DMEM to a final concentration of 1.5 × 10^5 fibroblasts/ml. Two parts of cell suspension were mixed with one part type 1 collagen (3 mg/ml) and neutralized with the appropriate amount of NaOH (defined by prior titration). 400 µl of the mixture was added per well of a 24-well plate. The plate was incubated for 30 min at 37 °C to let the collagen solidify. 400 µl DMEM 15% FBS was added on top of the gels and the gels were released from the edges with a pipette tip. After 24 h and images were taken without the lid at the Biorad ChemiDoc Imaging system in the Coomassie Blue channel with an exposure time of 0.2 s to document the size of the collagen gel.

### Graphical Representation and Statistical Analysis of RT-qPCR Results

For all statistical analysis and graphical displays of RT-qPCR results, the program GraphPad Prism (www.graphpad. com) was used. Relative mRNA levels/expression values of the four different sample categories were analysed through 1-way ANOVA (p-value with α = 0.05), followed by Bonferroni’s Multiple Comparison Test to assess significance between each of the categories. Significance is indicated with * = p < 0.05, ** = p < 0.01, *** = p < 0.001, **** = p < 0.0001 and ns = not significant. Data is displayed as scatter plots, with mean ± SEM.

## Electronic Supplementary Material

Below is the link to the electronic supplementary material.

Supplementary Material 1

Supplementary Material 2

Supplementary Material 3

Supplementary Material 4

Supplementary Material 5
