# Supplementary material for: Deciphering Stromal Changes between Metastatic and Non-metastatic Canine Mammary Carcinomas
Source: J Mammary Gland Biol Neoplasia. 2023 Jul 1;28(1):14. doi: 10.1007/s10911-023-09542-0 (PMC10313573; doi:10.1007/s10911-023-09542-0)
Supplement: Supplementary file 3 — Supplementary Material 3 [file 10911_2023_9542_MOESM3_ESM.docx]

| **metastatic cases** | | | | **non-metastatic cases** | | | |
| --- | --- | --- | --- | --- | --- | --- | --- |
| **Case #** | | **RIN** | **Total mRNA yield (ng)** | **Case #** | | **RIN** | **Total mRNA yield (ng)** |
| 1 | Normal stroma  CAS | 3.3  3.0 | 103.5  188.7 | 16 | Normal stroma  CAS | 2.4  3.4 | 198.9  60.3 |
| 2 | Normal stroma  CAS | 3.3  3.1 | 654  126.3 | 17 | Normal stroma  CAS | 2.8  1.1 | 147.3  244.8 |
| 3 | Normal stroma  CAS | 2.4  2.7 | 30.6  49.2 | 18 | Normal stroma  CAS | 2.8  3.0 | 52.5  109.8 |
| 4 | Normal stroma  CAS | 1.0  3.0 | 74.1  82.8 | 19 | Normal stroma  CAS | 3.0  3.8 | 56.4  103.5 |
| 5 | Normal stroma  CAS | 3.2  3.6 | 48.6  61.5 | 20 | Normal stroma  CAS | 3.3  3.5 | 43.5  63.9 |
| 6 | Normal stroma  CAS | 2.9  2.8 | 183.3  141.6 | 21 | Normal stroma  CAS | 2.8  3.1 | 110.1  111.9 |
| 7 | Normal stroma  CAS | 2.8  1.0 | 177  124.5 | 22 | Normal stroma  CAS | 3.3  3.7 | 35.4  39.3 |
| 8 | Normal stroma  CAS | 1.1  2.5 | 120  118.2 | 23 | Normal stroma  CAS | 2.6  3.1 | 83.7  62.4 |
| 9 | Normal stroma  CAS | 1.6  1.0 | 351  220.8 | 24 | Normal stroma  CAS | 2.4  2.9 | 135.9  81.3 |
| 10 | Normal stroma  CAS | 2.3  3.9 | 128.7  67.8 | 25 | Normal stroma  CAS | 2.9  3.8 | 62.1  48 |
| 11 | Normal stroma  CAS | 3.4  2.8 | 165  81.3 | 26 | Normal stroma  CAS | 3.4  2.4 | 51.6  202.5 |
| 12 | Normal stroma  CAS | 3.5  1.0 | 71.1  114 | 27 | Normal stroma  CAS | 3.1  2.8 | 51.6  59.1 |
| 13 | Normal stroma  CAS | 3.1  1.0 | 101.7  84.9 | 28 | Normal stroma  CAS | 3.3  3.3 | 31.5  37.2 |
| 14 | Normal stroma  CAS | 3.5  2.9 | 23.7  35.7 | 29 | Normal stroma  CAS | 3.3  3.7 | 49.2  30.6 |
| 15 | Normal stroma  CAS | 1.5  1.5 | 63  32.4 | 30 | Normal stroma  CAS | 4.0  3.5 | 33.3  26.9 |
|  |  |  |  | 31 | Normal stroma  CAS | 3.4  2.8 | 43.2  47.4 |

Ettlin *et al,* 2022

**Supplementary Table RNAvalues** Overview of RNA yields and RIN values of samples included in this study. RIN values and total yield of RNA are tabulated according to case# and type of tissue (normal stroma vs. CAS) in metastatic (left) versus non-metastatic (right) cases.
