# Supplementary material for: Deciphering Stromal Changes between Metastatic and Non-metastatic Canine Mammary Carcinomas
Source: J Mammary Gland Biol Neoplasia. 2023 Jul 1;28(1):14. doi: 10.1007/s10911-023-09542-0 (PMC10313573; doi:10.1007/s10911-023-09542-0)
Supplement: Supplementary file 4 — Supplementary Material 4 [file 10911_2023_9542_MOESM4_ESM.docx]

Ettlin *et al,* 2022

**Supplementary Table Primers**

List of Taqman primers used in this study for RT-qPCR of canine patient samples.

| **Gene** | **Sequence** | **Amplicon length (nt)** | **Taqman® order number or Reference** |
| --- | --- | --- | --- |
| TGFBR3 | Manufacturer’s proprietary information | 70 | Cf02637773_m1 |
| TGFB2 | Manufacturer’s proprietary information | 112 | Cf02676763_m1 |
| SFRP1 | Manufacturer’s proprietary information | 69 | Cf02654440_m1 |
| VIT | Manufacturer’s proprietary information | 68 | Cf02657286_m1 |
| MMP11 | Manufacturer’s proprietary information | 75 | ARFVK2U |
| GAPDH | Fw: 5’-GCTGCCAAATATGACGACATCA-3’  Rev: 5’-GTAGCCCAGGATGCCTTTGAG-3’  Probe: 5’-TCCCTCCGATGCCTGCTTCACTACCTT-3’ | 75 | [1] |
| B2M | Manufacturer’s proprietary information | 87 | Cf02659077_m1 |
| PPIA | Manufacturer’s proprietary information | 92 | Cf03986523_gH |

List of primers used in this study for RT-qPCR of human fibroblasts.

| **Gene** | **Sequence forward primer (5’ 🡪 3’)** | **Sequence reverse primer (5’ 🡪 3’)** |
| --- | --- | --- |
| **COL8A1** | AGAACTACAACCCGCAGAC | TTGAATAGAGCAACCCACA |
| **COL11A1** | TGGTGATCAGAATCAGAAGTTCG | AGGAGAGTTGAGAATTGGGAATC |
| **COL6A5** | AATCAGACGTGCCATCAACA | GGAGATGTTGTGCCTGGGAAT |
| **BGN** | GGTCTGAAGTCTGTGCCCAA | GAGCTCGGAGATGTCGTTGT |
| **GREM1** | CGGAGCGCAAATACCTGAAG | GGTTGATGATGGTGCGACTGT |
| **PCOLCE2** | TCGAGAGTGACAACCTGTGC | TGTTGCCACTGGACACAAGG |
| **SORCS2** | TGCTTCCCTTGAACCCTAAC | ACCGCGTTGTCACAGAAT |
| **LTBP4** | CGTCAGGCCACCTACACAG | CAGGGCCTCGAAGTCATCT |
| **VIT** | ATCTCTGGTCCACTGCCACCTA | ATCCGCTCCAACAGGTTTCTGG |
| **XRCC1** | TTGGAGAAGGAGGAGCAGAT | GGTGACCAGAAGGACCTCAT |
| **LRCC17** | AAAGTGCCAAACAACATCCCT | TGGGTCGAAGTTGGTTGATTTT |
| **SDK1** | GTCCGTGGGCTACAGGATTA | CATCTGCAGCTCGTATTCCA |
| **POSTN** | TGCCCAGCAGTTTTGCCCAT | CGTTGCTCTCCAAACCTCTA |
| **MMP11** | TCACCGAGAAGGGGATGTCC | TGGCCATATAGGTGTTGAAC |
| **B2M** | ATGTCTCGCTCCGTGGCCTTA | ATCTTGGGCTGTGACAAAGTC |
| **GAPDH** | AGCCACATCGCTCAGACAC | GCCCAATACGACCAAATCC |

**References:**

1. Amini P, Nassiri S, Malbon A, Markkanen E. Differential stromal reprogramming in benign and malignant naturally occurring canine mammary tumours identifies disease-modulating stromal components. Sci Rep. Nature Research; 2020;10.
