# Supplementary figures and images for: Deciphering Stromal Changes between Metastatic and Non-metastatic Canine Mammary Carcinomas
Source: J Mammary Gland Biol Neoplasia. 2023 Jul 1;28(1):14. doi: 10.1007/s10911-023-09542-0 (PMC10313573; doi:10.1007/s10911-023-09542-0)

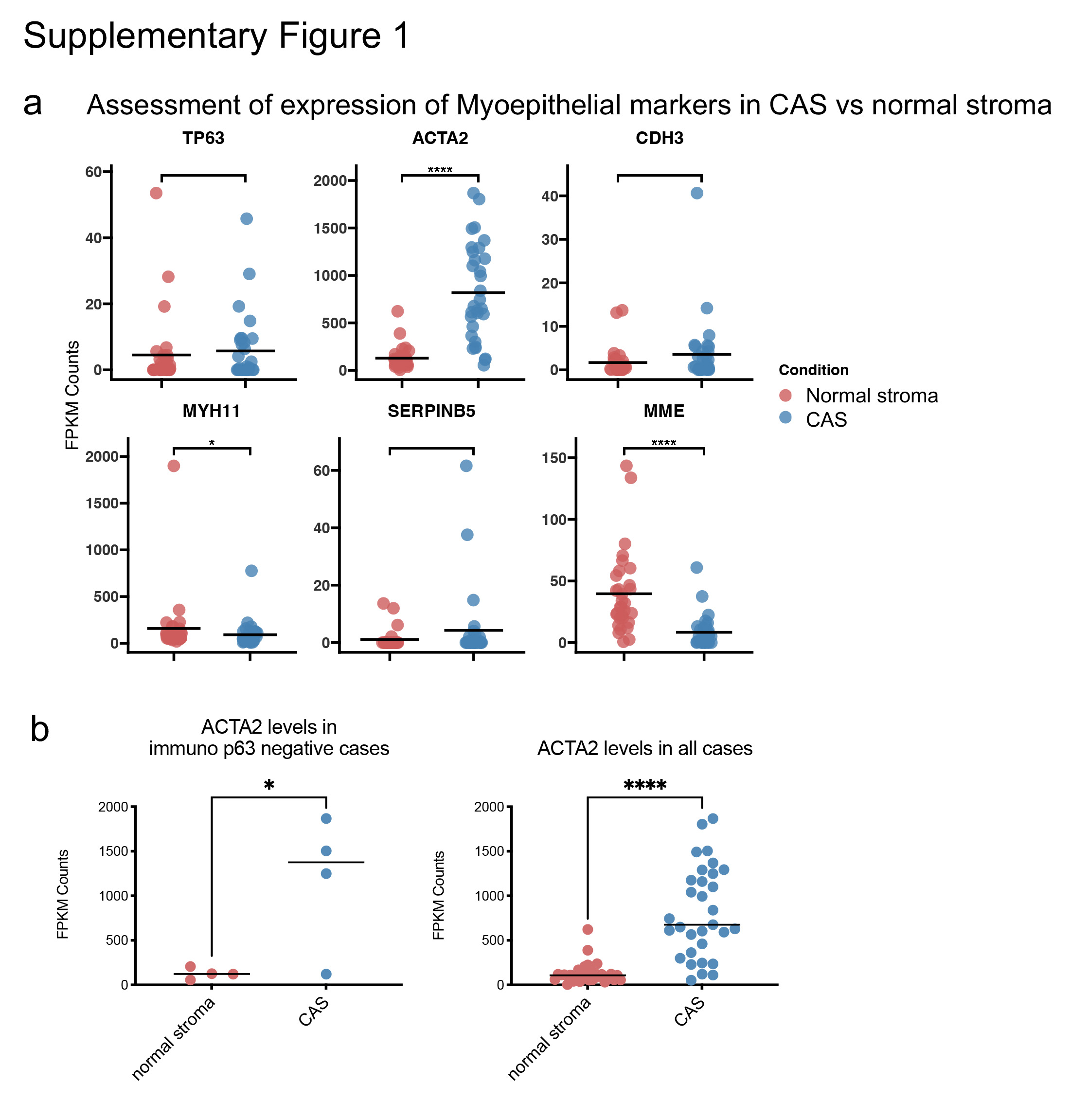

Supplement: Supplementary file 5 — Supplementary Material 5 [file 10911_2023_9542_MOESM5_ESM.jpg]
